# Breastfeeding and breastmilk substitute use and feeding motivations among mothers in Bandung City, Indonesia

**DOI:** 10.1111/mcn.13189

**Published:** 2021-04-16

**Authors:** Mackenzie Green, Alissa M. Pries, Dian N. Hadihardjono, Doddy Izwardy, Elizabeth Zehner, Victoria Hall Moran

**Affiliations:** ^1^ Helen Keller International Asia‐Pacific Regional Office Phnom Penh Cambodia; ^2^ Helen Keller International Headquarters Washington DC USA; ^3^ Helen Keller International Indonesia Country Office Jakarta Indonesia; ^4^ Kepala Pusat Penelitian, dan Pengembangan Upaya Kesehatan Masyarakat Badan Penelitian dan Pengembangan Kesehatan, Kementerian Kesehatan RI Jakarta Indonesia; ^5^ School of Community Health and Midwifery University of Central Lancashire Preston UK

**Keywords:** breastfeeding, breastmilk substitutes, infancy and childhood, infant feeding decisions, infant formula, International Code of Marketing of Breast‐milk Substitutes, marketing, policy

## Abstract

Suboptimal breastfeeding is common in Indonesia, with only half of infants 0–5 months of age exclusively breastfed and feeding of breastmilk substitutes (BMS) highly prevalent among infants and toddlers. Various factors influence these feeding practices, including social norms, limited health system support and BMS manufacturer marketing practices. This cross‐sectional survey aimed to identify the prevalence of breastfeeding and BMS feeding among children aged 0–35 months, explore socio‐demographic characteristics and motivating factors associated with these feeding behaviours and identify the prevalence of mothers' exposure to BMS promotions. Indonesian mothers of children <3 years of age (*n* = 595) were interviewed in Bandung City health facilities using structured questionnaires. Although all children were ever breastfed, half of children across all age groups received BMS in the previous day. Maternal employment outside the home and insufficient breastmilk production were associated with BMS use. The most important motivational factors for feeding BMS were perceived benefits for growth, intelligence and immunity. Despite Indonesian legislation restricting some BMS marketing, 93% of mothers reported observing a BMS promotion outside the health system, with television, social media and newspapers as the most common sources. Half of mothers (43%) reported observing a BMS promotion within the health system, and half (46%) reported receiving recommendations from health workers to use BMS. Such high prevalence of BMS marketing may be influencing caregivers' feeding choices; stronger national legislation and implementation of laws are needed to ensure mothers' ability to make feeding choices free from manufacturer influence.

**Key messages**

- Half of children across all ages received BMS in the previous day.
- BMS feeding was linked with mothers working outside the home and their perceived insufficient breastmilk production.
- The most important motivational factors for feeding BMS were perceived benefits on child growth, intelligence and immunity.
- Mothers' exposure to BMS promotions was widespread, and promotional health and nutrition claims may be misleading mothers.
- Full implementation of WHO recommendations on maternity protection and the International Code of Marketing of Breast‐milk Substitutes, including restricted promotion in the health system and of BMS products marketed for children <36 months, may facilitate improved breastfeeding practices in Indonesia.

## 1 INTRODUCTION

Breastfeeding is the single most effective preventative intervention to improve child survival (Jones et al., 2003). Life‐long benefits are conferred to the child, notably reducing risks of child mortality and morbidity and furthering child growth, development and cognitive achievement (Victora et al., 2016). The World Health Organization (WHO) recommends early initiation of breastfeeding within the first hour of life, exclusive breastfeeding for the first 6 months and continued breastfeeding to 2 years and beyond (World Health Organization [WHO] & UNICEF, 2003). Suboptimal breastfeeding practices lead to an estimated 823,000 preventable under‐five deaths annually (Victora et al., 2016) and USD 341 billion in global economic losses (Walters et al., 2019).

Multiple determinants and contexts lead to suboptimal breastfeeding practices. Deficient health service policies and practices leave mothers insufficiently supported or with incorrect information at critical breastfeeding timepoints (Rollins et al., 2016). Attitudes, preferences and cultural traditions of friends and family, including fathers, impact mothers' practices, as do those of employers and coworkers (Global Alliance for Improved Nutrition [GAIN], 2013; Rollins et al., 2016). Inadequate maternity protection policies, including for those in informal employment, lead to early cession of breastfeeding (Mason et al., 2013; Rollins et al., 2016). Personal attributes, like health status, education, weight and confidence, may sway feeding decisions (Rollins et al., 2016; Thulier & Mercer, 2009). Additionally, exposure to marketing of breastmilk substitute (BMS) products affects social norms on breastfeeding, undermines mothers' confidence and perceived self‐efficacy to breastfeed and influences attitudes on the safety and benefit of BMS (Piwoz & Huffman, 2015). Moreover, women living in resource‐poor areas can be more susceptible to BMS promotions as well as the risks posed by BMS consumption (Barennes et al., 2016).

The WHO established the International Code of Marketing of Breast‐milk Substitutes (the Code) (WHO, 1981) to protect mothers from unethical marketing of BMS products by manufacturers; however, adherence is often self‐regulated by manufacturers and dependent on national‐level legislation and enforcement (Baker et al., 2016; Rollins et al., 2016; WHO, 2020). Violations of the Code are commonplace in low‐ and middle‐income countries (LMIC) (Barennes et al., 2016; WHO, 2020), where national regulations and monitoring systems are not robust and the potential for corporate profit is high as their middle class expands, disposable income increases and greater numbers of women are entering the workforce (Baker et al., 2016; Mason et al., 2013; Rollins et al., 2016; Walters et al., 2016). Indonesia has one of the largest and most rapidly expanding BMS markets among all LMIC (Baker et al., 2016). Its substantial population and burgeoning middle class make it a lucrative market. In 2016, BMS sales topped IDR 34.3 billion (USD 2.5 billion), nearly doubling in value over the previous 5 years (Euromonitor International, 2016). Manufacturers engage in intense competition for market share, resulting in aggressive advertising and promotional activity.

Suboptimal breastfeeding practices are widespread in Indonesia (Beal et al., 2018; National Population and Family Planning Board [BKKBN] et al., 2018). Although nearly all children are ever breastfed, exclusive breastfeeding for infants 0–5 months was 51.5% nationally in 2017, and supplemental feeding with BMS is common (BKKBN et al., 2018). By 2 months of age, one in four breastfed children also receives BMS. At 6–23 months, 22.3% of breastfed and 72.9% of non‐breastfed children consume BMS. Although feeding with BMS may be medically necessary in some situations (WHO & UNICEF, 2009), sizeable evidence links partial or no breastfeeding in the first 6 months of life to poor health and development outcomes compared with exclusive breastfeeding (Black et al., 2008). Moreover, BMS is not advised or necessary after 12 months of age (Lott et al., 2019; WHO, 2013).

Few studies in Indonesia have documented mothers' exposure to BMS marketing and assessed the factors associated with BMS use among breastfeeding and non‐breastfeeding mothers of both infants and toddlers. To address this gap in the literature, this analysis explores the breastfeeding and BMS feeding practices of mothers of young children living in Bandung City, West Java. The primary objective was to identify the prevalence of breastfeeding and BMS feeding in mothers of children aged 0–35 months. Secondary objectives were to explore the influence of maternal and child characteristics on breastfeeding and BMS feeding; to explore the motivating factors that influence mothers to breastfeed and/or provide BMS; and to identify the prevalence of mothers' exposure to BMS marketing practices and recommendations to use BMS. This research contributes to the evidence base on BMS use and promotion in LMIC settings and builds understanding around the factors and motivations that may drive Indonesian mothers to use BMS. These findings can inform efforts to promote and protect optimal breastfeeding in Indonesia, as well as to renew national attention and action on regulating marketing and promotion of BMS.

## 2 METHODS

### 2.1 Study design, population and sampling

A cross‐sectional survey with multi‐stage cluster sampling was conducted from January to March 2018 in Bandung City, Indonesia, the fourth largest city in Indonesia and capital of West Java province (Badan Pusat Statistik [BPS] Kota Bandung, 2014). The sampling strategy for this survey was informed by WHO's NetCode protocol, which aims to assess prevalence of Code compliance (WHO & UNICEF, 2017). Women with children 0–35.9 months were recruited in health facilities to achieve a sample representative of mothers seeking child health services in Bandung City. Utilization of child health services is high in urban West Java; in 2012, 91% and 86% of 1‐year‐old children completed their DTP3 and measles vaccinations, respectively, and three‐quarters of under‐five children ill with respiratory infections or fever sought health care (Statistics Indonesia [BPS] et al., 2013). Therefore, health facilities were used as a proxy to reach the general population. Mothers were ineligible to participate if they lived outside Bandung City; their child was severely ill; they were not the biological mother; their child was from a multiple birth; they experienced severe delivery complications; or their child was in the neonatal intensive care unit. These factors may impede or delay breastfeeding and influence provision of BMS.

This analysis is part of a broader study to assess the use of commercial products for infant and young child feeding. Children 0–35 months were included as WHO's definition of BMS covers products marketed for children up to 3 years of age (WHO & UNICEF, 2017) and products intended for children 1 year and above are a rapidly growing market in Southeast Asia (Baker et al., 2020; Hastings et al., 2020). Sample size calculations for this present sub‐analysis were based on an estimated BMS consumption of 30% among 0‐ to 35‐month‐olds (Badan Pusat Statistik (BPS) Kota Bandung, 2014), a 0.05 alpha (Type I error) and 0.8 power (Type II error) and a design effect of 2 to account for cluster sampling, resulting in a sample of 253 mothers. Additional objectives of the broader study required a larger number of mothers (Green et al., 2019), and the final study sample size was 594.

Details on sampling, recruitment and data collection procedures for the broader study have been reported previously by Green et al. (2019). In summary, a list of the 60 public and private health facilities offering child health services in Bandung City was provided by the City Health Office. The number of under‐five child health visits to the 60 facilities in 2016 was collated by the study team and used to calculate the average number of child health visits per month per facility. Facilities with fewer than 100 visits per month (*n* = 17) were excluded from the sampling frame based on survey logistics. Facilities were then sampled through probability proportional to size, using average visits per month as the measure of size to allocate 33 clusters of 18 mothers each. The 18 mothers were recruited equally across 6‐month child age groups (0–5.9 months, 6.0–11.9 months, 12.0–17.9 months, 18.0–23.9 months, 24.0–29.9 months and 30.0–35.9 months), with three mothers per group per cluster.

A team of 10 interviewers, two recruiters and two supervisors were trained on ethics, questionnaire content and study procedures over 1 week in the classroom, followed by 1 week practicing in two unrelated health facilities. During data collection, every woman arriving for child health services at the facility was approached, screened and, if eligible, invited to participate. If the number of interviews for the child age‐group was complete for that facility, the mother would not be recruited.

Approval for this study was received from the Ethics Committee, Faculty of Medicine, Universitas Padjadjaran in Bandung. Written informed consent was obtained from all participants prior to interview.

### 2.2 Questionnaire design

Using a structured questionnaire, interviewers collected data on maternal age, parity, educational attainment and work outside of the home in the previous 1 month. The age and sex of the child were captured. A simplified subset of household asset questions was asked to assess household wealth status (EquityTool, 2019).

Data on breastfeeding and other liquids and foods consumed in the previous 24 h were collected according to WHO criteria for assessing infant and young child feeding (IYCF) practices (WHO, 2010). BMS was defined as formula, milk, or milk‐like products marketed for feeding children under 3 years of age (WHO & UNICEF, 2017). Mothers reporting BMS consumption in the prior day were asked to report consumption frequency in the previous week with three possible response options: (a) every day; (b) most days (4–6 days); and (c) about once a week (1–3 days) (Faber & Benadé, 2007). Mothers were also asked to provide the main reason why her child received BMS.

To assess factors motivating child feeding practices, mothers who provided breastmilk and/or BMS to their child in the previous day were asked to rate a series of reasons for breastfeeding and/or feeding BMS (Box 1). Mothers responded to a 4‐point Likert scale to rate how important each factor was for her personal decision in feeding her child, and options were *(1) not at all important; (2) not very important; (3) somewhat important; or (4) very important*. These close‐ended questions were adapted from the longitudinal Infant Feeding Practices Study II (Centers for Disease Control and Prevention [CDC], 2019) to capture prevalence rates of common motivations of breastfeeding versus BMS feeding and were pretested for comprehension with mothers in Bandung City prior to data collection. Mothers were also asked to identify which statement was closest to their opinion on the best way to feed a baby: (a) breastfeeding; (b) mix of both breastfeeding and formula feeding; (c) formula feeding; or (d) breastfeeding and formula feeding are equally good ways to feed a baby (CDC, 2019).

**BOX 1. Factors motivating mothers to breastfeed or provide BMS**

**Table:** 

**Breastfeeding factors**
My baby will have healthier/better immunity Breastfeeding supports my child's growth It will make my baby smart/intelligent It is what the health providers recommend that I should do It is what my relatives/friends believe I should do Breastfeeding is less expensive than feeding with breastmilk substitutes (saves money)
**Breastmilk substitute factors**
My baby will have healthier/better immunity with breastmilk substitutes Breastmilk substitutes support my child's growth It will make my baby smart/intelligent It is what the health providers recommend that I should do It is what my relatives/friends believe I should do I need to work I did not have enough breastmilk/my breastmilk did not satisfy my baby

Data were collected to determine if mothers had seen or heard a commercial promotion for BMS since the birth of their child and, if yes, to report the location of the promotion. A commercial promotion was defined as an advertisement, sign, display, free sample, gift, price discount, or other technique to induce purchase. Mothers were also asked to recall if, since the birth of their child, they had received a recommendation to use BMS, free samples of BMS, free samples of bottles or teats, or a branded gift. If a mother received a recommendation or any items, she was asked to name each source.

The questionnaire was translated into Bahasa Indonesia, back‐translated into English and pretested in two unsampled health facilities for clarity and accuracy. Data were collected with Samsung mobile tablets and the Open Data Kit (ODK) application. Completed questionnaires were uploaded nightly to an online data platform (ONA, 2019).

### 2.3 Variable creation and statistical analyses

Data were cleaned and analysed using Stata 14 (StataCorp, College Park, TX, USA). A wealth index was generated specific to the sample using principal components analysis and households were categorized into terciles of wealth (Green et al., 2019). Exclusive and predominant breastfeeding were calculated according to WHO IYCF indicators (WHO, 2010). Current breastfeeding was defined as the child receiving breastmilk in the previous day. A dichotomous variable was generated for the child's consumption of BMS in the previous day. Mothers were categorized into one of four groups based on current breastfeeding and consumption of BMS in the previous day: (a) Breastfeeding (BF), (b) Breastfeeding + BMS (BF + BMS), (c) BMS, (d) Neither. Categorization did not account for children's consumption of other liquids or foods in the previous day. A mean score was generated for each breastfeeding and BMS feeding motivating factor to assess at the population level the degree of importance of each factor in mothers' decision making. The score ranged from 1 to 4, with a higher score indicating stronger importance.

Exposure to BMS promotional activities was categorized to either inside the health system (i.e. hearing or seeing promotions inside any type of health facility or community health day) or outside the health system (i.e. hearing or seeing in all other locations). Recommendations to use BMS or receiving free sample or gifts from any type of health professional were categorized as inside the health system. Recommendations, free samples and free gifts from all other individuals were outside the health system; free samples and gifts from family, friends and other close acquaintances were excluded.

Percentage and number (n) were calculated for categorical variables and mean ± standard deviation (SD) for continuous variables. Differences in child consumption of breastmilk and BMS, main reason for feeding BMS and maternal exposure to BMS promotional activity by child age‐group were tested with logistic regression. Multinomial logistic regression was used to assess differences in maternal/child characteristics across the four groups of BF‐BMS feeding status. For this, binary variables were generated from each level of categorical independent variable of maternal/child characteristics. Differences in mean score for motivating factors by maternal characteristics and BF‐BMS feeding status groups were assessed using linear regression. To explore the interaction of child age with maternal characteristics and BF‐BMS feeding status on feeding motivators, age‐adjusted linear regression models were run with the same maternal characteristics, feeding status and feeding motivators along with continuous child age in months and the interaction of child age and maternal characteristic/feeding status. Statistical significance was defined as *p* < .05. All statistical tests controlled for clustering at the health facility level using Stata svy commands.

## 3 RESULTS

### 3.1 Sample characteristics, breastfeeding and BMS consumption

Among the 1440 mothers approached, 704 were eligible to participate. In total, 595 women completed surveys (84.5%), 21 had incomplete surveys (3.0%) and 88 refused to participate (12.5%), primarily due to lack of time. Green et al. (2019) have reported further on the sampling profile for facilities and mothers.

**FIGURE 1:**
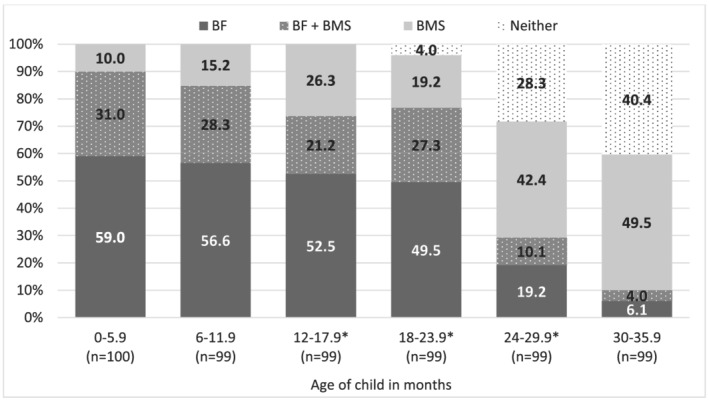
Percentage of children by breastfeeding‐BMS feeding status, across age groups (n=565). *p‐value < 0.01 difference between age‐group and next oldest age‐group. Significance testing was conducted using multinomial logistic regression adjusted for cluster at the facility level. BF‐BMS feeding status based on consumption in the previous day and does not account for consumption of other liquids or foods in the previous day. BF = breastfeeding; BF + BMS = breastfeeding and BMS feeding; BMS = BMS feeding; Neither = received neither breastfeeding nor BMS. Percentages may not add up to 100% due to rounding

Children ever breastfed was nearly universal (98.8%) in this sample. Figure 1 shows the consumption of breastmilk and BMS by child age‐group in the previous day. Most mothers with children under 2 years were currently breastfeeding (90.0% 0–5.9 months; 84.9% 6–11.9 months; 75.3% 12–23.9 months), while only 19.7% of children 24–35.9 months were currently breastfed (*p* < .001 difference between 24–35.9 months and each younger age group). Approximately half of all mothers reported feeding BMS in the previous day (*n* = 282; 47.4%) and the proportion providing BMS or BF + BMS was similar across all child age groups (41.0–53.5%) with no statistical differences.

Table 1 describes the maternal and child characteristics by BF‐BMS feeding status groups. Mothers of children 6–23.9 months of age who worked outside the home more commonly reported feeding their child BMS (33.3% 6–11.9 months; 35.6% 12–23.9 months) or BF + BMS (35.7% 6–11.9 months; 43.8% 12–23.9 months) compared to mothers who reported BF (12.5% 6–11.9 months, *p* = .034; 9.9% 12–23.9 months, *p* < .001). Higher educational attainment was associated with mothers feeding BMS, but only in mothers of older children. Half of mothers (54.2%) of 12‐ to 23.9‐month‐olds who consumed BMS and 42.2% of those who consumed BF + BMS had completed diploma or university‐level education, compared to 30.7% of BF mothers (*p* = .005). Similarly, 46.2% of BMS mothers with 24‐ to 35.9‐month‐olds had attained the highest level of education versus 32.0% of BF mothers and 21.4% of BF + BMS mothers (*p* = .001). Generally, household wealth did not differ among the four feeding status groups at any child age (data not shown).

**TABLE 1:** Maternal and child demographic characteristics by child breastfeeding‐BMS feeding status

Characteristic	All children (*n* = 595)	BF (*n* = 241)	BF + BMS (*n* = 121)	BMS (*n* = 161)	Neither (*n* = 72)	*p*‐value
Maternal age (years)	29.8 ± 5.6	29.3 ± 5.7	29.9 ± 5.7	30.5 ± 5.5	30.0 ± 5.3	.259
Primiparous	38.2 (227)	39.0 (94)	36.4 (44)	43.5 (70)	26.4 (19)	.067
Maternal education						
Elementary, junior high	20.8 (124)	23.7 (57)	15.7 (19)	17.4 (28)	27.8 (20)	.090
Senior high	43.4 (258)	44.0 (106)	42.2 (51)	39.8 (64)	51.4 (37)	.487
Diploma, university	35.8 (213)	32.4 (78)	42.2 (51)	42.9 (69)	20.8 (15)	.012
Maternal employment outside home in past month	22.0 (131)	14.5 (35)	33.9 (41)	27.3 (44)	15.3 (11)	<.001
Household wealth tercile						
Lowest wealth	32.9 (196)	37.8 (91)	27.3 (33)	25.5 (41)	43.1 (31)	.083
Middle wealth	31.1 (185)	27.8 (67)	33.1 (40)	35.4 (57)	29.2 (21)	.433
Highest wealth	36.0 (214)	34.4 (83)	39.7 (48)	39.1 (63)	27.8 (20)	.491
Child age (months)	17.3 ± 10.5	12.6 ± 8.4	12.8 ± 9.1	22.5 ± 9.5	29.4 ± 4.1	<.001
Child sex (male)	54.3 (323)	54.4 (131)	49.6 (60)	56.5 (91)	56.9 (41)	.424

*Note*: Data presented as percentage (*n*) or mean ± standard deviation. Percentages may not add up to 100% due to rounding. BF‐BMS feeding status is based on consumption in the previous day and does not account for consumption of other liquids or foods in the previous day. BF = breastfeeding; BF + BMS = breastfeeding and BMS feeding; BMS = BMS feeding; Neither = received neither breastfeeding nor BMS. Significance testing was conducted using multinomial logistic regression adjusted for cluster at the facility level.

Among infants under 6 months (*n* = 100), 46.0% were exclusively breastfed and 13.0% were predominantly breastfed. Non‐exclusively breastfed infants received BMS (75.9%), plain water (50.0%), juice/juice drinks (3.7%) and solid/soft foods (11.1%) in the previous 24 h. Over 99% of children 6 months and older had consumed solid foods in the day prior.

In those mothers who fed BMS in the previous day, weekly feeding frequency of BMS varied by whether the child received BMS alone or BF + BMS. Daily consumption was seen in nearly all children who received BMS alone (*n* = 161; 98.1%), irrespective of age (100% for 0–5.9, 6–11.9, and 12–23.9 months; 96.7% 24–35.9 months). Weekly feeding frequency among children receiving BF + BMS was less frequent (*n* = 121; 71.9% every day; 13.2% most days; 14.9% about once a week), and daily BMS consumption was lowest for infants 0–5.9 months (58.1%) compared with older children (71.4% 6–11.9 months; 79.2% 12–23.9 months; 78.6% 24–35.9 months).

### 3.2 Breastfeeding motivations

The most important factors motivating mothers to breastfeed (*n* = 362) were perceived benefits for child growth, health and immunity and child intelligence (Table 2). Health provider recommendations to breastfeed were rated more influential than those from family or friends. Across maternal characteristics and child age groups, there were no differences in scores for breastfeeding motivational factors. Mothers who practiced BF or BF + BMS placed a similar degree of importance for each of the six factors. There was no evidence to indicate that child age interacted with the maternal characteristics and breastfeeding motivators (Table S1).

**TABLE 2:** Mean score for factors motivating mothers to breastfeed by maternal characteristics and breastfeeding‐BMS feeding status

Characteristics	Breastfeeding motivational factors					
	Healthier/better immunity	Supports growth	Child smart/intelligent	Health providers recommend	Family and friends recommend	Saves money
All mothers breastfeeding (*n* = 362)	4.0 ± 0.1	4.0 ± 0.1	3.9 ± 0.3	3.8 ± 0.5	3.3 ± 0.8	3.6 ± 0.8
Maternal education						
Elementary, junior high (*n* = 76)	4.0 ± 0.2	4.0 ± 0.0	3.9 ± 0.3	3.9 ± 0.3	3.3 ± 0.8	3.6 ± 0.8
Senior high (*n* = 157)	4.0 ± 0.1	4.0 ± 0.2	3.9 ± 0.2	3.7 ± 0.6	3.2 ± 0.8	3.6 ± 0.8
Diploma, university (*n* = 129)	4.0 ± 0.1	4.0 ± 0.1	3.9 ± 0.3	3.8 ± 0.5	3.4 ± 0.9	3.6 ± 0.8
*p*‐value	.262	.531	.811	.001	.408	.998
Maternal employment						
Yes (*n* = 76)	4.0 ± 0.1	4.0 ± 0.2	3.9 ± 0.2	3.9 ± 0.4	3.5 ± 0.8	3.6 ± 0.7
No (*n* = 286)	4.0 ± 0.1	4.0 ± 0.1	3.9 ± 0.3	3.8 ± 0.5	3.3 ± 0.8	3.6 ± 0.8
*p*‐value	.946	.512	.732	.050	.056	.636
Household wealth tercile						
Low wealth (*n* = 124)	4.0 ± 0.2	4.0 ± 0.1	4.0 ± 0.2	3.8 ± 0.5	3.3 ± 0.8	3.6 ± 0.8
Middle wealth (*n* = 107)	4.0 ± 0.1	4.0 ± 0.1	4.0 ± 0.2	3.8 ± 0.5	3.3 ± 0.8	3.6 ± 0.7
High wealth (*n* = 131)	4.0 ± 0.1	4.0 ± 0.2	3.9 ± 0.3	3.8 ± 0.5	3.3 ± 0.9	3.6 ± 0.8
*p*‐value	0.412	0.538	0.398	0.947	0.749	0.786
Child age group (months)						
0–5.9 (*n* = 90)	4.0 ± 0.2	4.0 ± 0.1	4.0 ± 0.2	3.9 ± 0.4	3.4 ± 0.7	3.6 ± 0.8
6.0–11.9 (*n* = 84)	4.0 ± 0.2	4.0 ± 0.0	4.0 ± 0.2	3.8 ± 0.5	3.2 ± 0.9	3.5 ± 0.8
12.0–23.9 (*n* = 149)	4.0 ± 0.0	4.0 ± 0.1	3.9 ± 0.3	3.7 ± 0.6	3.3 ± 0.8	3.7 ± 0.7
24.0–35.9 (*n* = 39)	4.0 ± 0.0	3.9 ± 0.2	3.9 ± 0.2	3.7 ± 0.5	3.1 ± 0.8	3.6 ± 0.7
*p*‐value	.106	.036	.265	.046	.137	.508
BF‐BMS feeding status						
BF (*n* = 241)	4.0 ± 0.1	4.0 ± 0.1	4.0 ± 0.3	3.8 ± 0.5	3.3 ± 0.9	3.6 ± 0.8
BF + BMS (*n* = 121)	4.0 ± 0.2	4.0 ± 0.2	3.9 ± 0.3	3.7 ± 0.5	3.4 ± 0.8	3.6 ± 0.8
*p*‐value	.356	.165	.469	.396	.136	.651

*Note*: Data presented as mean ± standard deviation. Scores for each factor listed vertically in the table. BF‐BMS feeding status is based on consumption in the previous day and does not account for consumption of other liquids or foods in the previous day. BF = breastfeeding; BF + BMS = breastfeeding and BMS feeding; BMS = BMS feeding; Neither = received neither breastfeeding nor BMS. Significance testing was conducted using linear regression adjusted for cluster at facility‐level.

### 3.3 BMS motivations

All mothers who fed BMS (*n* = 282) reported that perceived benefits for their child, including growth, child intelligence and child health and immunity, were important factors for their decision to feed BMS (Table 3). Higher importance of these factors was negatively associated with educational attainment and wealth. Among working mothers, the need to work was a significantly important motivating factor. Mothers of the oldest children (24–35.9 months) were less motivated by insufficient production of breastmilk. The models to explore the interaction of age with maternal characteristics and BMS feeding motivators found no evidence to suggest there were differences by child age (Table S2), indicating the effect of the characteristics on motivation was constant.

**TABLE 3:** Mean score for factors motivating mothers to provide BMS by maternal characteristics and breastfeeding‐BMS feeding status

Characteristics	BMS feeding motivational factors						
	Healthier/better immunity	Supports growth	Child smart/intelligent	Health providers recommend	Family and friends recommend	Maternal work	Insufficient Breastmilk
All mothers feeding BMS (*n* = 282)	3.1 ± 0.9	3.4 ± 0.7	3.2 ± 0.9	3.0 ± 0.9	2.6 ± 0.9	2.6 ± 1.0	3.3 ± 0.9
Maternal education							
Elementary, junior high (*n* = 47)	3.3 ± 0.9	3.5 ± 0.7	3.6 ± 0.7	3.1 ± 1.0	2.7 ± 0.9	2.3 ± 1.2	3.4 ± 0.8
Senior high (*n* = 115)	3.1 ± 0.8	3.4 ± 0.7	3.3 ± 0.8	3.1 ± 0.9	2.7 ± 0.9	2.5 ± 1.0	3.3 ± 1.0
Diploma, university (*n* = 120)	2.9 ± 0.9	3.3 ± 0.7	2.9 ± 0.9	2.9 ± 1.0	2.5 ± 0.9	2.7 ± 1.0	3.3 ± 0.9
*p*‐value	.048	.041	.002	.321	.230	.042	.505
Maternal employment							
Yes (*n* = 85)	2.9 ± 0.9	3.3 ± 0.7	3.1 ± 0.9	2.9 ± 1.0	2.5 ± 0.8	3.3 ± 0.8	3.5 ± 0.8
No (*n* = 197)	3.1 ± 0.8	3.4 ± 0.7	3.2 ± 0.8	3.0 ± 0.9	2.6 ± 0.9	2.3 ± 1.0	3.2 ± 1.0
*p*‐value	.063	.164	.187	.403	.228	<.001	.029
Household wealth tercile							
Low wealth (*n* = 74)	3.1 ± 0.9	3.5 ± 0.7	3.5 ± 0.7	3.0 ± 1.0	2.6 ± 1.0	2.5 ± 1.0	3.4 ± 0.9
Middle wealth (*n* = 97)	3.1 ± 0.8	3.4 ± 0.7	3.3 ± 0.8	3.1 ± 0.9	2.7 ± 0.9	2.6 ± 1.1	3.2 ± 0.9
High wealth (*n* = 111)	3.0 ± 0.9	3.3 ± 0.7	2.9 ± 0.9	2.9 ± 0.9	2.5 ± 0.8	2.5 ± 1.0	3.4 ± 0.9
*p*‐value	.474	.279	.006	.149	.128	.830	.363
Child age group (months)							
0–5.9 (*n* = 41)	2.8 ± 0.8	3.2 ± 0.7	3.4 ± 0.7	2.9 ± 1.0	2.7 ± 0.9	2.6 ± 1.0	3.4 ± 0.9
6.0–11.9 (*n* = 43)	2.8 ± 0.8	3.2 ± 0.9	3.1 ± 0.9	3.1 ± 0.9	2.7 ± 0.9	2.8 ± 1.0	3.5 ± 0.7
12.0–23.9 (*n* = 93)	3.1 ± 0.9	3.4 ± 0.7	3.2 ± 0.9	3.0 ± 1.0	2.5 ± 0.9	2.6 ± 1.1	3.6 ± 0.7
24.0–35.9 (*n* = 105)	3.3 ± 0.8	3.5 ± 0.6	3.2 ± 0.9	3.0 ± 0.9	2.6 ± 0.9	2.4 ± 1.0	3.0 ± 1.1
*p*‐value	.008	.027	.403	.635	.857	.340	.003
BF‐BMS feeding status							
BMS (*n* = 161)	3.2 ± 0.9	3.5 ± 0.7	3.2 ± 0.9	3.1 ± 0.9	2.6 ± 0.9	2.4 ± 1.0	3.4 ± 0.9
BF + BMS (*n* = 121)	2.8 ± 0.8	3.2 ± 0.7	3.1 ± 0.8	2.9 ± 1.0	2.6 ± 0.9	2.8 ± 1.0	3.3 ± 0.9
*p*‐value	.001	.001	.407	.028	.369	.021	.272

*Note*: Data presented as mean ± standard deviation. Scores for each factor listed vertically in the table. BF‐BMS feeding status is based on consumption in the previous day and does not account for consumption of other liquids or foods in the previous day. BF = breastfeeding; BF + BMS = breastfeeding and BMS feeding; BMS = BMS feeding; Neither = received neither breastfeeding nor BMS. Significance testing was conducted using linear regression adjusted for cluster at facility‐level.

Mothers' motivations to feed BMS varied based on their BF‐BMS feeding status. Among mothers feeding BMS only, the perceived benefits of BMS to their child's health/immunity and growth were scored significantly higher compared with mothers who provided BF + BMS, though the small differences in scores may not be meaningful. The mothers who fed BF + BMS (*n* = 121) consistently ranked better immunity, growth and child intelligence as more important factors in their decision to breastfeed than their decision to provide BMS. Recommendations from health care providers and friends and family were also considered more important to mothers' decision to feed breastmilk than to feed BMS.

The main reason for feeding BMS in the previous week was perceived milk insufficiency (i.e. ‘I did not have enough breastmilk’), reported by 41.6% and 43.8% of mothers in the BMS and BF + BMS groups, respectively (*p* = .73); followed by perceived health benefits of BMS (i.e. ‘They are healthy/good for the child's development’), which were reported by 29.2% and 18.2% of mothers, respectively (*p* = .055). In both BMS‐feeding groups, the proportion of mothers citing health benefits of BMS increased with child age (BMS: 0%, 6.7%, 20.0%, 40.7%, for increasing child age‐groups, *p* = .027; BF + BMS: 12.9%, 17.9%, 12.5%, 50.0%, *p* = .006), whereas those reporting insufficient breastmilk had an inverse relationship with child age (BMS: 90.0%, 80.0%, 53.3%, 24.2%, *p* < .001; BF + BMS: 61.3%, 42.9%, 41.7%, 14.3%, *p* = .010). A greater proportion of BF + BMS mothers cited needing to work as their main reason compared with BMS mothers (17.4% vs. 5.0%, *p* = .005); however, there were no significant differences by child age‐group.

Mothers reporting these top three reasons—insufficient breastmilk, health benefits and need to work—also scored the highest on the corresponding BMS motivational factor reinforcing it to be the most important factor in their decision to feed BMS (3.7 ± 0.6 for insufficient breastmilk, 3.6 ± 0.6 for perceived benefit on child growth and 3.6 ± 0.6 for maternal work, respectively).

### 3.4 Opinion on best feeding practice

Two‐thirds (68.6%) of all mothers believed the best way to feed a baby is ‘Breastfeeding’, whereas one‐quarter (24.5%) aligned with the statement ‘Breastfeeding and formula feeding are equally good ways to feed a baby’. When examined by BF‐BMS feeding status (Table 4), very few mothers considered BMS to be superior, even among those providing BMS alone. Opinions did not differ significantly by maternal characteristics or child age group.

**TABLE 4:** Percentage of mothers reporting their opinion of the best infant/child feeding practice by their breastfeeding‐BMS feeding status across age groups

Opinion on best way to feed a baby	BF (*n* = 241)	BF + BMS (*n* = 121)	BMS (*n* = 161)	Neither (*n* = 72)	*p*‐value
‘Breastfeeding’	80.9 (195)	56.2 (68)	52.8 (85)	83.3 (60)	<.001
‘Mix of both breastfeeding and formula feeding’	4.6 (11)	7.4 (9)	8.1 (13)	1.4 (1)	.379
‘Formula feeding’	0.4 (1)	0.0 (0)	3.1 (5)	1.4 (1)	<.001
‘Breastfeeding and formula feeding are equally good ways to feed a baby’	14.1 (34)	36.4 (44)	36.0 (58)	13.9 (10)	<.001

*Note*: Data presented as percentage (*n*). Percentages may not add up to 100% due to rounding. BF‐BMS feeding status is based on consumption in the previous day and does not account for consumption of other liquids or foods in the previous day. BF = breastfeeding; BF + BMS = breastfeeding and BMS feeding; BMS = BMS feeding; Neither = received neither breastfeeding nor BMS. Significance testing was conducted using multinomial logistic regression adjusted for cluster at facility‐level. Overall, *p*‐value is <.001.

### 3.5 BMS promotional activity

Exposure to commercial BMS promotions since the birth of their child was nearly ubiquitous among all women (93.3%). Mothers of children over 1 year were more likely to report exposure (96.7% 12–23.9 months; 98.0% 24–35.9 months) than mothers of infants 0–5.9 months (83.0%; *p* = .004 and *p* = .001, respectively) and mothers of infants 6–11.9 months (86.9%; *p* = .002 and *p* < .001). Most mothers observed promotions outside of the health system (93.1%), but 42.9% reported exposure to them inside the health system. Promotions outside the health system (*n* = 554) were predominantly observed in media (97.8%), including television (94.2%), social media (60.7%), and magazines and newspapers (37.7%). Mothers also observed promotions in retail locations (87.2%), billboards (53.4%), and with company representatives (37.9%).

Almost half of all mothers (45.7%) received a recommendation to use BMS since the birth of their child, with no difference by child age. Nearly a quarter of all mothers (*n* = 135; 22.7%) reported receiving a recommendation within the health system, including from a doctor (34.8%), nurse (31.9%) or midwife (44.4%). Just under one‐third of all mothers (*n* = 172; 28.9%) reported receiving a recommendation outside the health system, which came mainly from family (76.2%). More mothers who used BMS received a recommendation to feed BMS from inside the health system (31.7% BMS and 24.8% BF + BMS) compared with BF mothers (16.5%) or Neither (19.4%; *p* = .017).

Since the birth of their child, 26.1% of all mothers reported receiving free samples of BMS. By child age group, 8.0% of mothers of the youngest infants (0–5.9 months) said they received BMS samples compared with a quarter or more of mothers of older children (26.8% 12–23.9 months, *p* = .002; 39.9% 24–35.9 months, *p* = .001). Free BMS samples were more commonly received outside the health system (17.1%) compared with inside the health system (9.8%). Few of the mothers (5.7%) reported receiving free samples of feeding bottles/teats, and none reported receiving branded gifts.

## 4 DISCUSSION

This cross‐sectional survey among mothers of children under 3 years living in Bandung City, Indonesia, found widespread use of BMS, which has previously been undocumented for children 24–36 months in Indonesia. BMS use for young child feeding was associated with maternal work and perceptions of insufficient breastmilk production; however, the most important maternal motivations for feeding BMS were the perceived benefits on child growth, intelligence and immunity. With mothers reporting near universal exposure to BMS promotions, it is plausible that health and nutrition claims for BMS products may be influencing caregivers' feeding choices and contributing to suboptimal breastfeeding practices in Indonesia, although these associations require further study.

Similar to recent national data, 98.8% of children in our study were breastfed at some point in infancy (96% nationally) and 46.0% of those aged up to 6 months of age were exclusively breastfed (51.1% nationally) (BKKBN et al., 2018). Among children who were not exclusively breastfed, three‐quarters received BMS and half received plain water. Non‐exclusive breastfeeding during the first 6 months is a risk factor for stunting and infant mortality due to several preventable diseases, including diarrhoea. The prevalence of child stunting remains high in Indonesia, at around 37%, and diarrhoea is a leading cause of child mortality, with over a third of all children under‐five in the country seeking treatment for the condition (Beal et al., 2018; UNICEF, 2020). BMS products risk microbial contamination and improper preparation (Baker et al., 2016; Barennes et al., 2016; Mason et al., 2013), exacerbated by poor availability of safe drinking water in the home; around one‐fifth of the Indonesian population only has access to ‘unimproved’ drinking water sources (i.e. surface water, unprotected dug wells and unprotected springs) (Patunru, 2015). One study of children under 2‐years in East Java found 88% of BMS feeds had high levels of bacterial contamination and were ‘unfit for human consumption’ (Gibson et al., 2017). Early cessation of exclusive breastfeeding has been estimated to contribute to 5377 preventable infant deaths per year in Indonesia due to diarrheal and respiratory disease with an annual cost to the healthcare system of USD 119 million per year (1.6 trillion Rupiah) (Siregar et al., 2018; Walters et al., 2016).

BMS use was common across all age groups in our study. Although most mothers of children 0–23 months reported they were breastfeeding, nearly half also fed their child BMS. BMS feeding for children under 2 years was associated with mothers working outside the home. Mothers' employment has been linked to reduction in exclusive and less frequent breastfeeding in LMIC (Agunbiade & Ogunleye, 2012; Lakati et al., 2002; Lesorogol et al., 2018). A systematic review of factors influencing breastfeeding exclusivity during the first 6 months found a strong negative association of formal employment or work outside the home with exclusive breastfeeding (Balogun et al., 2015). In‐depth interviews with mothers in Ghana (Otoo et al., 2009) and Tanzania (Shao Mlay et al., 2004) describe how short maternity leaves or lack of on‐site feeding locations may partially explain this relationship. Current national legislation in Indonesia supports 3 months maternity leave, of which 1.5 months are taken antenatally and 1.5 months are taken following birth (Blaney et al., 2014). Although public policy states that employers should provide a suitable place for mothers to breastfeed their infants or express milk, previous research in Indonesia reported that compliance with existing policies and legislation in support of exclusive breastfeeding is inconsistent and where available, some women may feel that breastfeeding or breastmilk pumping at work is inappropriate (Flaherman et al., 2018; Siregar et al., 2019). Expanding maternity leave, ensuring the availability of workplace lactation facilities and encouraging support by peers will help safeguard breastfeeding among working mothers (Basrowi et al., 2018; Siregar et al., 2018).

The use of growing‐up milks or toddler milks—BMS products intended for children 12–35 months of age—was prevalent among nearly half of 1–3 year olds, with the majority consuming them daily. The use of growing‐up milks has been increasing globally and is a burgeoning market for manufacturers (Baker et al., 2020; Hastings et al., 2020). In Indonesia, Euromonitor International (2016) reports 40% volume growth in sales of infant and young child milk formula over 2011–2016, with growing‐up milks rising most substantially. WHO and expert panels recommend against the use of growing‐up milks (Lott et al., 2019; WHO, 2013), which provide no unique nutritional value and are ultra‐processed products composed primarily of powdered milk, vegetable oil and sweeteners (Lott et al., 2019). An investigation of growing‐up milks in Indonesia found 98% contained one or more added sugars/sweeteners, and few products met global requirements for sugar content and composition (Helen Keller International, 2020). Exposure to sweet‐tasting beverages early in life capitalizes on infants' innate preference for sweet tastes (Ventura & Worobey, 2013) and has been associated with establishing sweet taste preferences throughout later childhood (Luque et al., 2018). Breastfeeding into the second year of life and beyond provides continued morbidity protection and confers unique contributions to the diets of young children (Sankar et al., 2015).

Exposure to BMS promotions both outside and inside the health system was commonplace in our study and reinforces previous findings of widespread violations of the Code in Indonesia (Durako et al., 2016; GAIN, 2013; Hadihardjono et al., 2019; Hidayana et al., 2017; Nuzrina et al., 2016; Roshita et al., 2013; Shetty, 2014; Susiloretni et al., 2015; Vinje et al., 2017). Inadequate enforcement of breastfeeding‐related legislation is observed in many LMIC and likely contributes substantially to suboptimal breastfeeding practices in Indonesia (Siregar et al., 2019). Marketing of BMS targets mothers and health workers which undermines women's confidence and disincentivises breastfeeding (Piwoz & Huffman, 2015; Shetty, 2014). Although Indonesia is one of the 136 of 194 WHO member states that has enacted legal measures to implement the Code, its current measures are only ‘moderately aligned’ with WHO's guidance (WHO, 2020). In particular, it has limited measures to protect against engagement with health workers and promotion of BMS to the general public. In our study, mothers feeding BMS were more likely to have a health worker recommend BMS compared with breastfeeding mothers, a trend witnessed in other LMIC (Champeny et al., 2019; Rothstein et al., 2020). Exposure to commercial BMS promotions was nearly universal in our study, and recent research documented rampant promotion of BMS in retail locations throughout Bandung City (Hadihardjono et al., 2019). Over three‐quarters of available growing‐up milks were promoted in stores, which is allowable under Indonesian legislation but violates the Code. Aligning infant food and beverage marketing regulations with global standards will strengthen protection of the breastfeeding mother (Hadihardjono et al., 2019). Restrictions on marketing must be expanded from 12 to 36 months and a system for monitoring and reporting violations, including strong penalties for violators, should be established, coupled with the exclusion of the formula industry from nutrition, education and policy roles (Barennes et al., 2016; Hadihardjono et al., 2019).

Although most women in our study were aware that breastfeeding was optimal for their baby, a quarter of those surveyed believed breastfeeding and formula feeding were equally good ways to feed their babies. Other studies in Indonesia also show that despite widespread recognition that breastfeeding is best, many women worry that breastfeeding alone, without BMS supplementation, is insufficient (Euromonitor International, 2016; GAIN, 2013). Given that breastfeeding is promoted by the Indonesian Ministry of Health and the use of child health services in Bandung City is high, it is striking that one quarter of our respondents held this incorrect belief. Indonesian regulations permit products for children aged 1–3 years to make nutrient content claims, and a recent study in Indonesia found that almost all growing‐up milks did in fact make nutrient content claims on their labels, including statements around high micronutrient content (Helen Keller International, 2020). With almost all women in our study reporting exposure to BMS promotion, it is plausible that these promotions and the nutrition claims made for growing‐up milks may be misleading some consumers into believing these products are equally as good as breastmilk.

Commercial promotions may also be influencing Indonesian mothers' perceptions of the apparent health and developmental benefits of BMS. All mothers feeding BMS in this study ranked the perceived benefits of child growth, intelligence and health and immunity as the strongest motivators in their decision to feed BMS. These perceived benefits are strategic marketing messages employed by the formula industry to imply their product ingredients support brain development and strengthen immunity in young children (Harris & Pomeranz, 2020; Romo‐Palafox et al., 2020) and have been documented in Indonesia (Euromonitor International, 2016; Hastings et al., 2020). There is limited scientific evidence to substantiate these claims (Hughes et al., 2017), and one recent study demonstrated that caregivers who agreed with these health and development marketing claims had increased odds of feeding BMS to their child (Romo‐Palafox et al., 2020). Further research on the impact of health and nutrient content claims is needed within contexts like Indonesia where BMS use is prevalent.

Many women also cited perceived insufficient breastmilk as a reason for feeding their children BMS. Lack of knowledge, confidence and self‐efficacy have been widely reported as reasons among mothers for less than optimum breastfeeding duration (GAIN, 2013; Thulier & Mercer, 2009), and perceptions of insufficient milk are commonly cited for early introduction of complementary feeding or not breast‐feeding exclusively (Bunik et al., 2010; Kent et al., 2020) including in Indonesia (GAIN, 2013; Nuzrina et al., 2016; Roshita et al., 2013). Women's perception of milk insufficiency may stem from anxiety about her own nutritional status or meeting her infant's nutritional needs and infant satiety, advice from and role modelling of family members about mixed feeding, insufficient support from the health system and perceived infant feeding norms (GAIN, 2013; Safon et al., 2017; Susiloretni et al., 2015). Interventions to improve breastfeeding education, self‐efficacy and/or support have been shown to increase exclusive breastfeeding rates and decrease no breastfeeding rates up to 6 months of age and may be particularly effective in LMICs (Galipeau et al., 2018; Haroon et al., 2013). Such interventions have the potential to improve optimum breastfeeding practices and should be scaled up, although there remains a paucity of evidence on the mode, format and intensity needed to provide optimal outcomes.

Our study has some limitations. First, our research was limited to an urban area of one of Indonesia's 34 diverse provinces. Given its geographic and population size and its social, economic and cultural diversity, further research is needed to determine whether similar issues are found in other areas of the country. Second, our health facility‐based survey may yield potential bias in our sample. There may be differences between mothers who seek child health services versus mothers who do not utilize these services. However, as noted, health seeking behaviours are highly prevalent in West Java province and we are confident that this sample is representative of the majority of the Bandung City population. Due to the use of a health facility‐based design for this survey, a substantial proportion of children in our sample (60%) were reported sick the previous day, which may impact feeding practices. Additionally, our survey did not collect data on maternal attributes that may impact breastfeeding practices, like health or experience with trauma/violence, as they were beyond the scope of this assessment. Nonetheless, our study found breastfeeding and BMS rates that were comparable to large demographic survey data and so may be representative of the wider population. We also noted no difference in the proportion of children that were sick the previous day across age groups, with the exception of a lower rate among 0‐ to 5.9‐month‐olds (40% as compared with 53–68% among older age groups). Third, we defined BMS consumption according to whether the child had consumed it on the previous day, which would have excluded those that fed BMS more infrequently and therefore we may have underestimated the proportion of children receiving BMS. In addition, data on exposure to promotional activity and recommendations are subject to recall bias and mothers with older children had longer periods for possible exposure given that mothers were asked about promotions observed since the birth of their child. Finally, we found that the age of the child had no influence on mothers' motivations for feeding their children breastmilk or BMS, but our age‐adjusted models may not have been sufficiently powered to fully explore this interaction.

## CONFLICT OF INTEREST

The authors have no conflicts of interest to declare.

## CONTRIBUTIONS

MG and VHM prepared the manuscript. MG analysed the data, with input from AMP. AMP and EZ designed the study with input from MG, DH and DI. MG oversaw questionnaire development. Enumerator training was overseen by DH, MG and AMP; DH supervised data collection; and MG oversaw data management. All authors reviewed and provided inputs on the final article.

## Supporting information


**Table S1.** Results of age‐adjusted linear regression models to assess the interaction of child age (months) and maternal characteristics on breastfeeding motivational factors

**Table S2.** Results of age‐adjusted linear regression models to assess the interaction of child age (months) and maternal characteristics on BMS feeding motivational factors

## Data Availability

The data that support the findings of this study are available from the corresponding author upon reasonable request.
