# Supplementary material for: Breastfeeding and breastmilk substitute use and feeding motivations among mothers in Bandung City, Indonesia
Source: Matern Child Nutr. 2021 Apr 16;17(3):e13189. doi: 10.1111/mcn.13189 (PMC8189241; doi:10.1111/mcn.13189)
Supplement: Supplementary file 1 — Table S1. Results of age‐adjusted linear regression models to assess the interaction of child age (months) and maternal characteristics on breastfeeding motivational factors [file MCN-17-e13189-s002.docx]

Table S1. Results of age-adjusted linear regression models to assess the interaction of child age (months) and maternal characteristics on breastfeeding motivational factors

|  | Breastfeeding motivational factors | | | | | | |
| --- | --- | --- | --- | --- | --- | --- | --- |
| Characteristics | Healthier/ Better Immunity | Supports Growth | Child Smart/ Intelligent | Health Providers Recommend | Family and Friends Recommend | Saves Money |  |
| Maternal education |  |  |  |  |  |  |  |
| Interaction coefficient | -0.0021 | -0.0004 | 0.0025 | 0.0008 | -0.0072 | -0.0094 |  |
| Coefficient P-value | 0.171 | 0.429 | 0.280 | 0.793 | 0.157 | 0.047 |  |
| Overall model P-value | 0.449 | 0.065 | 0.703 | 0.001 | 0.224 | 0.398 |  |
| Maternal employment |  |  |  |  |  |  |  |
| Interaction coefficient | 0.0001 | -0.0004 | 0.0005 | 0.0047 | 0.0004 | -0.0045 |  |
| Coefficient P-value | 0.950 | 0.807 | 0.868 | 0.465 | 0.971 | 0.699 |  |
| Overall model P-value | 0.153 | 0.162 | 0.680 | 0.032 | 0.200 | 0.572 |  |
| Household wealth tercile |  |  |  |  |  |  |  |
| Interaction coefficient | -0.0012 | 0.0003 | -0.0001 | 0.0009 | 0.0001 | -0.0078 |  |
| Coefficient P-value | 0.152 | 0.767 | 0.944 | 0.833 | 0.991 | 0.133 |  |
| Overall model P-value | 0.372 | 0.175 | 0.522 | 0.190 | 0.618 | 0.549 |  |
| BF-BMS feeding status |  |  |  |  |  |  |  |
| Interaction coefficient | 0.0017 | -0.0001 | 0.0014 | 0.0059 | 0.0014 | 0.0086 |  |
| Coefficient P-value | 0.417 | 0.952 | 0.707 | 0.267 | 0.883 | 0.299 |  |
| Overall model P-value | 0.252 | 0.036 | 0.363 | 0.028 | 0.293 | 0.518 |  |

Note: Coefficients and P-values for each factor listed vertically in table. Age-adjusted models run using linear regression adjusted for cluster at facility-level. Models include motivational factor, characteristic, child age (continuous months), interaction of characteristic and child age.
