# Supplementary material for: Breastfeeding and breastmilk substitute use and feeding motivations among mothers in Bandung City, Indonesia
Source: Matern Child Nutr. 2021 Apr 16;17(3):e13189. doi: 10.1111/mcn.13189 (PMC8189241; doi:10.1111/mcn.13189)
Supplement: Supplementary file 2 — Table S2. Results of age‐adjusted linear regression models to assess the interaction of child age (months) and maternal characteristics on BMS feeding motivational factors [file MCN-17-e13189-s001.docx]

Table S2. Results of age-adjusted linear regression models to assess the interaction of child age (months) and maternal characteristics on BMS feeding motivational factors

|  | | BMS feeding motivational factors | | | | | | |  |
| --- | --- | --- | --- | --- | --- | --- | --- | --- | --- |
| Characteristics | Healthier/ Better Immunity | | Supports Growth | Child Smart/ Intelligent | Health Providers Recommend | Family and Friends Recommend | Maternal Work | Insufficient Breastmilk | |
| Maternal education |  | |  |  |  |  |  |  | |
| Interaction coefficient | 0.0093 | | 0.0055 | 0.0060 | 0.0041 | -0.0007 | -0.0166 | -0.0020 | |
| Coefficient P-value | 0.145 | | 0.379 | 0.431 | 0.643 | 0.902 | 0.058 | 0.761 | |
| Overall model P-value | 0.002 | | 0.011 | 0.006 | 0.446 | 0.479 | 0.005 | 0.074 | |
| Maternal employment |  | |  |  |  |  |  |  | |
| Interaction coefficient | -0.0088 | | -0.0047 | 0.0137 | -0.0003 | 0.0109 | -0.0116 | 0.0188 | |
| Coefficient P-value | 0.379 | | 0.558 | 0.230 | 0.980 | 0.456 | 0.328 | 0.135 | |
| Overall model P-value | 0.001 | | 0.001 | 0.280 | 0.575 | 0.431 | <0.001 | 0.007 | |
| Household wealth tercile |  | |  |  |  |  |  |  | |
| Interaction coefficient | 0.0043 | | 0.0016 | 0.0026 | -0.0012 | 0.0006 | -0.0115 | -0.0079 | |
| Coefficient P-value | 0.379 | | 0.749 | 0.637 | 0.823 | 0.925 | 0.130 | 0.274 | |
| Overall model P-value | 0.001 | | 0.011 | 0.037 | 0.260 | 0.251 | 0.151 | 0.030 | |
| BF-BMS feeding status |  | |  |  |  |  |  |  | |
| Interaction coefficient | 0.0061 | | 0.0155 | 0.0063 | 0.0072 | -0.0123 | -0.0065 | 0.0100 | |
| Coefficient P-value | 0.464 | | 0.084 | 0.603 | 0.635 | 0.364 | 0.598 | 0.508 | |
| Overall model P-value | 0.001 | | <0.001 | 0.354 | 0.181 | 0.674 | 0.151 | 0.001 | |

Note: Coefficients and P-values for each factor listed vertically in table. Age-adjusted models run using linear regression adjusted for cluster at facility-level. Models include motivational factor, characteristic, child age (continuous months), interaction of characteristic and child age.
